# Public assessment of key performance indicators of healthcare in a Canadian province: the effect of age and chronic health problems

**DOI:** 10.1186/2193-1801-3-28

**Published:** 2014-01-15

**Authors:** Abu Sadat Nurullah, Herbert C Northcott, Michael D Harvey

**Affiliations:** Department of Sociology, University of Alberta, 5-21 Tory Building, Edmonton, Alberta T6G 2H4 Canada; Edmonton, Alberta Canada

**Keywords:** Key performance indicators, Healthcare system, Age group, Chronic illness, Chronic pain, Alberta

## Abstract

This study explores the effect of age and chronic conditions on public perceptions of the health system, as measured by the Key Performance Indicators (KPIs) of healthcare, in the province of Alberta in Canada. Drawing from data collected by Government of Alberta’s Department of Health and Wellness, this research examines two key questions: (1) Do people in the 65+ age group rate the KPIs of healthcare (i.e., availability, accessibility, quality, outcome, and satisfaction) more favorably compared to people in younger age groups in Alberta? (2) Does the rating of KPIs of healthcare in Alberta vary with different chronic conditions (i.e., no chronic problem, chronic illnesses without pain, and chronic pain)? The findings indicate that people in the older age group tend to rate the KPIs of healthcare more favorably compared to younger age groups in Alberta, net of socio-demographic factors, self-reported health status, and knowledge and utilization of health services. However, people experiencing chronic pain are less likely to rate the KPIs of healthcare favorably compared to people with no chronic health problem in Alberta. Discussion includes implications of the findings for the healthcare system in the province.

**Electronic supplementary material:**

The online version of this article (doi:10.1186/2193-1801-3-28) contains supplementary material, which is available to authorized users.

## Introduction

Performance indicators of healthcare are important measures of public confidence in and satisfaction with the healthcare system. Furthermore, patients can provide valuable information about key aspects of healthcare (Draper et al., 2001). Pelone et al. (2008) suggested that performance indicators of healthcare should include assessments of effectiveness, technical efficiency, accessibility, equity of access, timeliness, and safety. Performance indicators foster the use of patients’ views for identifying problems and difficulties encountered in health services and gaps in the quality of care, and thus allow health authorities to formulate better strategies and management procedures for the healthcare system (Mpinga and Chastonay, 2011). Therefore, at present, there is a growing research interest in patients’ perceptions of their care (Infante et al., 2004).

Previous research on public perceptions of healthcare includes the effects of general health status, chronic conditions (e.g., arthritis, chronic back and neck pain), mental health conditions (e.g., depression), and socio-demographic factors on various performance indicators (e.g., availability, accessibility, utilization, quality, and satisfaction). These studies on patient assessment of their care often report inconsistent findings.

### Patients’ age and assessment of healthcare

Existing research suggests that older patients, on average, are more likely to be satisfied with healthcare services that they have received compared to younger patients, and this is consistent across cultures and nations (Bleich et al., 2009; Campbell et al., 2001; Cohen, 1996; Crow et al., 2002; Hall and Dornan, 1990; Lyratzopoulos et al., 2012; Moret et al., 2007; Quintana et al., 2006; Rahmqvist, 2001; Rahmqvist and Bara, 2010; Sixma et al., 1998; Sofaer and Firminger, 2005; Tucker and Kelley, 2000; Young et al., 2000). Studies have suggested that older patients are more satisfied because they may be more unwilling to criticize the service they receive and may be more tolerant compared to younger patients (Agoritsas et al., 2009; Hall and Dornan, 1990). Jayasinghe et al. (2008) suggested that patients who are younger may have higher expectations of the service, and when those expectations are not met, they may tend to assess the service negatively. However, a few studies found either a negative association or no significant relationship between age and patient satisfaction (Boudreaux et al., 2000; Jaipaul and Rosenthal, 2003; Kane et al., 1997; Moret et al., 2007).

Other socio-demographic characteristics such as patients’ sex, education, income, ethnicity, marital status, family size, living condition (i.e., living alone or living with others) etc. failed to show a consistent trend in predicting patient satisfaction because findings were often contradictory (Crow et al., 2002; Glynn et al., 2004; Hall and Dornan, 1990; Quintana et al., 2006; Rahmqvist and Bara, 2010; Sofaer and Firminger, 2005; Upmark et al., 2007; Zhang et al., 2007).

Previous research indicated that positive experience related to *access* to healthcare increased with age, with older patients being more satisfied with access to care compared to younger ones (Jayasinghe et al., 2008; Kontopantelis et al., 2010; Wilson and Rosenberg, 2004). In addition, older age was related to greater utilization of medical services (Field and Briggs, 2001; Pappa and Niakas, 2006). Some studies have suggested that some older patients may not accurately recall their difficulty in accessing healthcare services in the past 12 months due to cognitive impairment (Kasman and Badley, 2004; Raina et al., 2002).

### Chronic health conditions and assessment of healthcare

People experiencing chronic health conditions such as chronic pain spend more days in hospital or other care facilities, and are more in need of medical care than those who do not have chronic conditions (Millar, 1996). Chronic health problems affect most aspects of one’s life ranging from work to social relationships (Millar, 1996). Studies have showed consistently that factors associated with increased prevalence of chronic pain include female sex, increasing age, being divorced or separated, higher body mass index, poor self-reported health, and indicators of lower socioeconomic status, such as less education, low income, being unemployed, and residence in public housing (Boulanger et al., 2007; Johannes et al., 2010; Millar, 1996; Moulin et al., 2002; Ramage-Morin and Gilmour, 2010; Reitsma et al., 2011; Schopflocher et al., 2011; Tripp et al., 2006).

Researchers have suggested that people with chronic pain are regular users of a variety of healthcare services and are more likely to assess their self-reported health negatively than individuals without chronic pain (Ramage-Morin and Gilmour, 2010; Tripp et al., 2006). Chronic pain is associated with sleep deficiency, activity and mobility limitations, cognitive impairment, other chronic diseases, anxiety, social withdrawal, loneliness, depression, negative affects (e.g., a tendency to view the world as threatening), loss of self-confidence and self-esteem, and poor physical and mental health (Millar, 1996; Ohayon and Schatzberg, 2010; Ramage-Morin, 2008; Toblin et al., 2011; Tsang et al., 2008). Constraint on leading a normal life was identified as the main problem of chronic pain (Smith et al., 1999). People with one or more chronic conditions were more likely than those without a chronic condition to report not receiving healthcare when required (Kontopantelis et al., 2010; Wilson and Rosenberg, 2004). Furthermore, patients with multiple chronic illnesses reported a higher level of hassles in accessing health care system compared to patients with a single chronic illness (Parchman et al., 2005). A study conducted by Bentur et al. (2004) found that chronic illness was associated with longer wait times for an appointment with a specialist.

Self-reported health status in general influences public assessment of healthcare. Studies have shown that satisfaction scores are higher in those patients who had better self-reported overall health (Cohen, 1996; Crow et al., 2002; Hall et al., 1990; Jaipaul and Rosenthal, 2003; Rahmqvist and Bara, 2010; Sofaer and Firminger, 2005; Wendt et al., 2009; Xiao and Barber, 2008). Previous studies generally suggest that patients who are in poor health tend to be less satisfied with the care that they receive (Al-Mandhari et al., 2004; Bleich et al., 2009; Crow et al., 2002; Glynn et al., 2004; Hall et al., 1999; Lyratzopoulos et al., 2012; Schoenfelder et al., 2011; Tucker, 2002; Wensing et al., 1997; Zhang et al., 2007). In a longitudinal sample of patients aged 70 and above, Hall et al. (1993) tested the causal pathways between satisfaction and health status, and found that self-perceived good health was related to more satisfaction a year later, but not vice versa. Similarly, in a longitudinal study, Ren et al. (2001) found that patients with better health status — particularly better mental health status — were more satisfied with their hospital care. It has also been suggested that poor health in general may directly produce dissatisfaction in patients (Hall et al., 1998). However, a few studies found that health status was not significantly related to satisfaction (Bertakis et al., 1991; Soh, 1991). Finally, research suggests that since healthier patients are inclined to report greater satisfaction with health care, it is health status per se, rather than *degree of improvement* in health status because of medical care, that affects patients’ satisfaction scores (Rapkin et al., 2008).

With regard to access to care, studies found that self-reported good health (vs. poor health) was associated with better access to healthcare services (Glynn et al., 2004; Jayasinghe et al., 2008; Põlluste et al., 2012; de Boer et al., 2010). Jayasinghe et al. (2008) suggested that patients with better health may need fewer visits for hospital care, and hence have less chance of experiencing difficulty in access to care.

### Objectives of the current study

The aforementioned review of literature suggests that although a plethora of research has investigated patients’ perception about a particular indicator of healthcare (e.g., satisfaction), past studies rarely examine the combined impact of multiple indicators of healthcare in a general population. Most of these studies focus on patients in different situations (inpatient facility, outpatient, emergency care, suffering from diverse disease conditions, etc.) and across different socio-demographic characteristics. In addition, only a handful of studies examined the specific expectations of individuals with chronic conditions (Infante et al., 2004), particularly chronic pain. Furthermore, this line of research is lacking in the context of Alberta. In order to address these research gaps, the current study examines two questions: (1) Do people in the older age group score the KPIs of healthcare (i.e., availability, accessibility, quality, and satisfaction) higher on average compared to people in younger age groups in Alberta? and (2) Does the rating of KPIs of healthcare in Alberta vary with different chronic conditions (i.e., no chronic problem, chronic illnesses without pain, and chronic pain)?

## Methods

### Data and sample

The dataset used in this analysis comes from the *2004 Public Survey about Health and the Health System in Alberta*. This annual survey was launched in 1996 by the Department of Health and Wellness of the Government of Alberta and continued in similar format until 2004 (Northcott and Northcott, 2004). A cross-sectional, representative sample of 4,000 adults (age 18 and above) from the nine health regions of Alberta participated in the 2004 survey. The survey was conducted by the Population Research Laboratory at the University of Alberta and data were collected by telephone (random digit dialing using computer assisted telephone interviewing) from February 10 to March 31, 2004. The original survey was approved by the Research Ethics Board of the University of Alberta. The sample was stratified based on age, sex, and health region of the participants. The response rate for the 2004 survey was 72%.

### Measures

#### Dependent variable

The dependent variable for this study is an index of key performance indicators (KPIs) of the healthcare system in Alberta. These indicators reveal a single underlying construct measuring public perceptions of healthcare (see Northcott and Harvey, 2012 for details). The KPIs index consists of five general indicators assessing the availability, accessibility, and perceived quality of healthcare services, overall quality of the healthcare system, and satisfaction with the health system in Alberta. Four of these KPIs were measured on a 4-point scale and one (satisfaction) on a 5-point scale. Availability was measured by asking the respondents, “Overall, how would you rate the *availability* of health care services in your community?” (coded 1 = poor, 4 = excellent). Accessibility was measured by the question, “How easy or difficult is it for you to get the health care services you need when you need them?” (coded 1 = very difficult, 4 = very easy). Perceived quality of the healthcare services was measured by the question, “Overall, how would you rate the *quality* of health care services that are available in your community?” (coded 1 = poor, 4 = excellent). Quality of the healthcare system was measured by the question, “Thinking now about the health care system in Alberta, overall, how would you rate it?” (coded 1 = poor, 4 = excellent). Satisfaction with the health system was measured by the question, “Overall, how satisfied are you with the health system in Alberta?” (coded 1 = very dissatisfied, 5 = very satisfied). Northcott and Harvey (2012) utilized the same sample used in this study and reported a Cronbach’s alpha value of .84 for the KPIs index, indicating good internal consistency for this measure.

#### Independent variables

Age and chronic conditions are examined in this study as possible predictors of the KPIs, that is, of assessments of the healthcare system in Alberta. In the original survey, age was divided into five categories: 18–24, 25–44, 45–64, 65–74, and 75+ years. For this analysis, *age* is coded as ‘non-seniors’ (18 to 64) = 0 and ‘seniors’ (65+) = 1. *Chronic condition* was classified into three categories: no chronic problem, at least one chronic condition (e.g., neurological diseases, heart and circulatory diseases, asthma and other chronic respiratory diseases, diabetes and other endocrine diseases, cancer, genito-urinary, reproductive, allergies, muscular or skeletal, sensory system) excluding chronic pain, and chronic pain with or without other chronic conditions.

#### Control variables

Self-reported general health status, self-reported physical and mental health status in past 30 days, knowledge of the available health services, utilization of healthcare services, and socio-demographic characteristics were controlled in this study. *Self-reported general health status* was measured by the question, “In general, compared with other people your age, would you say your health is…” (coded 1 = poor, 5 = excellent). *The physical and mental health status in past 30 days* were continuous measures (number of days) comprising two questions: “Thinking of your physical health, which includes physical illness and injury, for how many days during the past 30 days was your physical health *not* good?” and “Thinking of your mental health, which includes stress, depression, and problems with emotions, for how many days during the past 30 days was your mental health *not* good?” *Knowledge of the available health services* was measured by the question, “In general, how would you rate your knowledge of the health services that are available to you?” (coded 1 = poor, 4 = excellent). *Utilization of healthcare services* was measured by the question, “Have you *personally* received any health care services in Alberta in the past 12 months?” (coded 0 = no, 1 = yes).

#### Demographic and socioeconomic characteristics

Demographic and socioeconomic indicators included sex, education, household income, and living arrangement. Sex was coded as male = 0, female = 1. Education was classified into four categories: less than high-school, completed high-school, some post-secondary, and completed post-secondary. Annual household income before taxes and deductions was classified into four categories: less than $30,000, $30,000 to $59,999, $60,000 to 99,999, and $100,000+. Living arrangement was coded as 0 = living alone, 1 = living with someone.

### Analysis

The analysis was carried out using IBM SPSS version 20 (PASW) and included univariate, bivariate, and multivariate analysis of data involving computation of percentages, ANOVA, and hierarchical multiple regression. Association among age, chronic conditions, and the KPIs was tested using ANOVA. In order to identify the significant predictors of the KPIs, multivariate ordinary least squared (OLS) regression analyses were performed. Initial analyses were performed on each separate KPIs, which showed that the findings were significant (results not shown and are available upon request). This was followed by analyses of combined KPIs index in three models. Data were checked to ensure no violation of the assumption of normal distributions (Tabachnick and Fidell, 2007). There were no problems of multicollinearity as the highest VIF (variance inflation factor) score was 3.05 (Cohen et al., 2003: 423). Sampling weights were applied for all analyses in this study (based on the population distribution across the health regions) so as to provide a representative sample of adult Albertans (see Northcott and Northcott, 2004, pp. 53–54). Because household income is missing for approximately 22% of the cases, a separate regression analysis was conducted excluding income from the models (not shown here). However, it did not produce a significant difference in outcome. Therefore, the final analysis includes income.

## Results

Table 1 presents descriptive findings for the sample, dependent variable, independent variables and control variables. Females represented 50.4% and males represented 49.6% of the sample. A majority of the sample (86.2%) were non-seniors while 13.8% were seniors (*n* = 552). About one half of the respondents had completed post-secondary education (49.9%), and had an annual household income of $60,000 and above (51.2%) before taxes and deductions. In terms of household arrangement, 15.2% of the respondents were living alone while 84.8% were living with other members of the household.

**Table 1:** **Sample, independent variable and dependent variable characteristics from the 2004 Alberta Health survey**

Variables		***N***	% or***Mean***	***SD***	Adjusted %
Sex					
	Male	1984	49.6		49.6
	Female	2016	50.4		50.4
Age					
	Non-Seniors (18–64)	3448	86.2		86.2
	Seniors (65–75+)	552	13.8		13.8
Education					
	Less than high-school	525	13.1		13.2
	Completed high-school	926	23.2		23.3
	Some post-secondary	619	15.5		15.6
	Completed post-secondary	1902	47.5		49.9
	*Missing*	28	0.7		
Annual household income					
	Up to $29,999	591	14.8		19.0
	$30,000 to $59,999	928	23.3		29.8
	$60,000 to $99,999	862	21.5		27.6
	$100,000+	734	18.3		23.6
	*Missing*	886	22.1		
Living arrangement					
	Living alone	607	15.2		15.2
	Living with someone	3388	84.7		84.8
	*Missing*	5	0.1		
Chronic conditions					
	No chronic problem	2836	70.9		71.1
	Chronic without pain	1059	26.5		26.5
	Chronic pain	97	2.4		2.4
	*Missing*	8	0.2		
Self-reported health status			*3.64*	*1.03*	
	Poor	140	3.5		3.5
	Fair	391	9.8		9.8
	Good	1080	27.0		27.0
	Very good	1523	38.0		38.1
	Excellent	862	21.6		21.6
	*Missing*	3	0.1		
Days in past 30 days physical health not good			*3.84*	*8.24*	
	0 day	2406	60.2		60.7
	1–7 days	1024	25.6		25.8
	8+ days	536	13.4		13.5
	*Missing*	33	0.8		
Days in past 30 days mental health not good			*3.31*	*7.20*	
	0 day	2461	61.5		62.1
	1–7 days	1003	25.1		25.3
	8+ days	500	12.5		12.6
	*Missing*	36	0.9		
Knowledge of the available health services			*2.72*	*0.82*	
	Poor	302	7.6		7.6
	Fair	1146	28.7		28.9
	Good	1870	46.7		47.1
	Excellent	652	16.3		16.4
	*Missing*	30	0.8		
Personally received healthcare services					
	No	895	22.4		22.4
	Yes	3100	77.5		77.6
	*Missing*	6	0.1		
Availability of healthcare services			*2.60*	*0.88*	
	Poor	497	12.4		12.8
	Fair	1084	27.1		27.8
	Good	1783	44.6		45.8
	Excellent	531	13.3		13.6
	*Missing*	106	2.6		
Accessibility of healthcare services			*2.69*	*0.83*	
	Very difficult	292	7.3		7.5
	A bit difficult	1222	30.6		31.3
	Easy	1778	44.4		45.4
	Very easy	616	15.4		15.8
	*Missing*	91	2.3		
Quality of healthcare services			*2.78*	*0.81*	
	Poor	283	7.1		7.3
	Fair	945	23.6		24.3
	Good	2005	50.1		51.6
	Excellent	654	16.4		16.8
	*Missing*	112	2.8		
Quality of healthcare system in Alberta			*2.67*	*0.77*	
	Poor	303	7.6		7.7
	Fair	1087	27.2		27.7
	Good	2115	52.9		53.9
	Excellent	419	10.5		10.7
	*Missing*	75	1.9		
Satisfaction with the health system in Alberta			*3.74*	*0.99*	
	Very dissatisfied	139	3.5		3.5
	Somewhat dissatisfied	427	10.7		10.8
	Neither	474	11.8		12.0
	Somewhat satisfied	2208	55.2		55.9
	Very satisfied	705	17.6		17.8
	*Missing*	47	1.2		
Key Performance Indicators (KPIs) Index			*2.90*	*0.67*	
	Total cases	3719	93.0		
	*Missing*	281	7.0		

Note: *N* = 4,000. The Mean and Standard Deviation values are shown in italics.

A large number of respondents (71.1%) had no chronic health problem that would require regular health services, 26.5% had one or more chronic conditions without chronic pain, and 2.4% (*n* = 97) had chronic pain with or without other chronic conditions. The majority of the respondents (86.7%) reported that their general health was good, very good or excellent. During the past 30 days, 39.3% and 37.9% of the respondents reported that their physical and mental health, respectively, were *not* good. When asked about their knowledge of the health services that were available to them, 63.5% rated their knowledge as either good or excellent. A substantial proportion of respondents (77.6%) reported personally receiving healthcare service(s) in the province during the past year.

In terms of the availability of healthcare services in their community, 59.4% rated availability as either good or excellent. A little less than two-thirds of the respondents (61.2%) reported that it was either easy or very easy for them to access healthcare services when they needed them. Approximately two-thirds of the respondents rated the quality of available healthcare services and the overall healthcare system in Alberta as either good or excellent (68.4% and 64.6%, respectively). A majority of the respondents (73.7%) reported that they were either somewhat or very satisfied with the healthcare system in the province.

**Figure 1:**
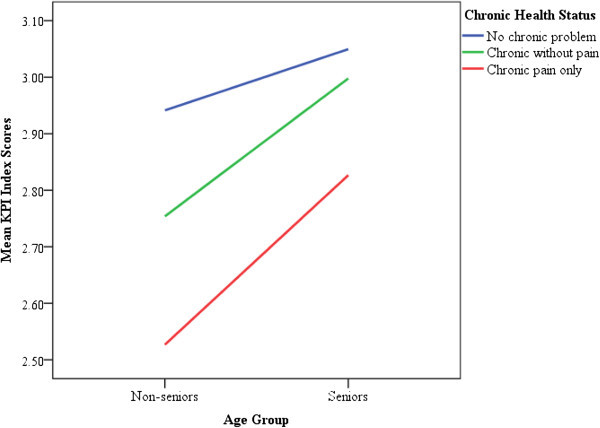
**Mean scores of KPI Index comparing chronic conditions by age group.**

Because seniors are more likely to rate the KPIs more favorably (Bleich et al., 2009; Campbell et al., 2001; Field and Briggs, 2001; Hall and Dornan, 1990; Kontopantelis et al., 2010; Pappa and Niakas, 2006; Quintana et al., 2006; Rahmqvist and Bara, 2010; Sofaer and Firminger, 2005; Wilson and Rosenberg, 2004; Young et al., 2000) and because the respondents’ chronic conditions are negatively related to their perceptions on the healthcare system (Jayasinghe et al., 2008; Ramage-Morin and Gilmour, 2010; Schoenfelder et al., 2011; Sofaer and Firminger, 2005; Tripp et al., 2006), it was necessary to assess the possible interaction effect of age and chronic conditions on the KPIs using a two-way ANOVA test. Figure 1 illustrates that the interaction was not statistically significant, *F* (2, 3813) = 2.102, *p* = .122.

Multivariate ordinary least squared (OLS) regression models were used to estimate the predictors of the KPIs of the healthcare system in Alberta. Age and chronic conditions were entered at step 1, other socio-demographic indicators were entered at step 2, self-reported health statuses and knowledge about and utilization of healthcare services were entered at step 3, resulting in 3 predictive models (see Additional file 1 for coding of the variables). As illustrated in Model 1 of Table 2, age (*β* = 0.114, *p* < .001) and chronic conditions [chronic without pain, *β* = -0.114, *p* < .001, and chronic pain, *β* = -0.104, *p* < .001] significantly predicted the KPIs assessing the healthcare system, *F* (3, 2865) = 29.04, *p* < .001. Moreover, the inclusion of socio-demographic variables in Model 2 did not significantly alter the coefficient values of age and chronic conditions.

**Table 2:** **Unstandardized and standardized beta coefficients of key performance indicators of the health system in Alberta**

		Model 1		Model 2		Model 3	
Variables		***b***	***β***	***b***	***β***	***b***	***β***
Chronic health problem status							
	No chronic problem (Ref)		—		—		—
	Chronic without pain	-0.175 (0.03)	-0.114***	-0.165 (0.03)	-0.107***	-0.067 (0.03)	-0.044*
	Chronic pain	-0.451 (0.08)	-0.104***	-0.435 (0.08)	-0.100***	-0.232 (0.08)	-0.054**
Age							
	Non-seniors (Ref)		—		—		—
	Seniors	0.238 (0.04)	0.114***	0.248 (0.04)	0.119***	0.130 (0.04)	0.063***
Sex							
	Male (Ref)				—		—
	Female			-0.075 (0.03)	-0.056*	-0.096 (0.02)	-0.072***
Education							
	Less than high-school (Ref)				—		—
	Completed high-school			0.069 (0.05)	0.043	0.035 (0.04)	0.022
	Some post-secondary			0.037 (0.05)	0.020	-0.002 (0.05)	-0.001
	Completed post-secondary			-0.001 (0.04)	-0.001	-0.089 (0.04)	-0.066*
Annual household income							
	Up to 29,999 (Ref)				—		—
	$30,000 to $59,999			-0.029 (0.04)	-0.020	-0.052 (0.04)	-0.035
	$60,000 to $99,999			-0.018 (0.04)	-0.012	-0.071 (0.04)	-0.048
	$100,000+			-0.031 (0.04)	-0.020	-0.094 (0.04)	-0.060*
Living arrangement							
	Living alone (Ref)				—		—
	Living with someone			0.068 (0.04)	0.036	0.057 (0.04)	0.030
Self-reported health status						0.049 (0.01)	0.074***
Days physical health not good							
	0 day (Ref)						—
	1–7 days					-0.024 (0.03)	-0.016
	8+ days					-0.129 (0.04)	-0.065**
Days mental health not good							
	0 day (Ref)						—
	1–7 days					-0.091 (0.03)	-0.060***
	8+ days					-0.293 (0.04)	-0.145***
Knowledge of available health services						0.177 (0.02)	0.213***
Personally received healthcare services							
	No (Ref)						—
	Yes					0.063 (0.03)	0.039*
Constant			2.947***		2.921***		2.384***
Δ *F*			29.038***		2.377*		71.911***
*R* ^2^			0.030		0.036		0.119
Adj. *R* ^2^			0.028		0.032		0.113
Δ *R* ^2^			—		0.006*		0.044***

Note: Weighted *N* = 2,870. Standard errors are in parentheses.

*p < .05, **p < .01, ***p ≤ .001 (two-tailed tests).

Ref = Reference category.

As illustrated in Model 3, having any chronic health problem excluding chronic pain (*β* = -0.044, *p* = .033) and having chronic pain negatively predicted the KPIs (*β* = -0.054, *p* = .004) assessing healthcare in Alberta. In addition, being in the senior age group positively (*β* = 0.063, *p* = .001) predicted the KPIs. In other words, relative to those with no chronic condition, those with one or more chronic problems excluding chronic pain more negatively assessed the KPIs and those with chronic pain assessed the KPIs even more negatively. On the other hand, senior participants more positively evaluated the KPIs compared to those who were non-seniors. Sex predicted the KPIs such that compared to males, the coefficient for females was negatively associated with the KPIs (*β* = -0.072, *p* < .001). Participants’ higher education (*β* = -0.066, *p* = .032) and higher annual household income (*β* = -0.060, *p* = .025) were negatively associated with the KPIs. However, the living arrangement of the participants was not a significant predictor of the KPIs (*β* = 0.030, *p* = .114). Participants’ self-reported health status (*β* = 0.074, *p* = .001), and knowledge (*β* = 0.213, *p* < .001) and utilization of healthcare services (*β* = 0.039, *p* = .036) positively predicted the KPIs. Participants’ poor physical health status during the past 30 days (8+ days) negatively predicted the KPIs (*β* = -0.065, *p* = .002). Finally, participants’ mental health status during the past 30 days (1 to 7 days and 8+ days, respectively) negatively predicted the KPIs (*β* = -0.060, *p* = .001, and *β* = 0.145, *p* < .001, respectively). Overall, knowledge of healthcare services and mental health status during the past 30 days were the strongest predictors of the KPIs (based on their respective standardized regression coefficients). The adjusted *R*^2^ value for model 3 was 0.119, indicating that 12% of the variance in the KPIs of healthcare was explained by the model.

## Discussion and conclusions

This research examined the effects of age and chronic illness on Albertan’s perceptions of their health system, as measured by the KPIs of healthcare. Regarding the first objective of the study, the results indicated that people in the older age group rated the KPIs of healthcare (i.e., availability, accessibility, quality, and satisfaction) more positively compared to the younger age group in Alberta, after taking into account socio-demographic factors, self-reported health status, and knowledge and utilization of health services. This is consistent with previous studies conducted in Canada and elsewhere that showed older people were more likely to be satisfied with their received healthcare services and reported better access to care compared to those who were young (Bleich et al., 2009; Campbell et al., 2001; Cohen, 1996; Hall and Dornan, 1990; Jayasinghe et al., 2008; Kasman and Badley, 2004; Lyratzopoulos et al., 2012; Rahmqvist and Bara, 2010; Rahmqvist, 2001; Sofaer and Firminger, 2005; Tucker and Kelley, 2000; Wilson and Rosenberg, 2004; Young et al., 2000). This association seems to persist irrespective of healthcare delivery settings (i.e., inpatient care, emergency care, ambulatory, and private clinic visits) (Scotti, 2005).

Existing literature suggested several factors that may influence older people to positively assess the KPIs of healthcare. It could be that older people have more exposure to the health system and therefore have more pragmatic expectations of their care compared to younger ones (Hordacre et al., 2005). Another potential reason is that older individuals may be more unwilling to criticize the service they receive compared to younger ones (Agoritsas et al., 2009; Hall and Dornan, 1990). In addition, it has been suggested that some older patients may not accurately recall their difficulty in accessing the healthcare services in the past 12 months due to cognitive impairment (Kasman and Badley, 2004; Raina et al., 2002). However, it was not possible with the cross-sectional data used in this study to determine the reason for older people’s more favorable rating of the KPIs of healthcare. As such, future research should explore this issue using longitudinal design that allows establishing cause and effect.

For the socio-demographic controls, the findings indicated that females rated the KPIs of healthcare more negatively compared to males, after controlling for possible covariates. This is in agreement with previous studies suggesting that women are more likely than men to report experiencing negative encounters with healthcare (Cohen, 1996; Quintana et al., 2006; Upmark et al., 2007), or difficulty in accessing healthcare (Kasman and Badley, 2004). It could also be that women suffer more from chronic health problems (including back pain and neck pain), depression, and related illnesses compared to men (Boulanger et al., 2007; Malmusi et al., 2011; Moulin et al., 2002; Reitsma et al., 2011; Schopflocher et al., 2011; Tsang et al., 2008), and as a result women may tend to rate the KPIs more negatively. Furthermore, this study found that respondents’ higher education and higher income were marginally and negatively associated with their assessment of the KPIs of healthcare in Alberta, which is consistent with the findings of previous studies (Hekkert et al., 2009; Jayasinghe et al., 2008; Quintana et al., 2006; Rahmqvist and Bara, 2010; Sahin et al., 2006; Sofaer and Firminger, 2005).

With reference to the second objective of this study, the findings revealed that people experiencing chronic pain and other chronic illnesses rated the KPIs of healthcare more negatively compared with people who had no chronic health problem in Alberta. This is in line with previous studies reporting that patients experiencing severe pain and/or chronic illness are more likely to be dissatisfied with the care they receive (Cohen, 1996; Crow et al., 2002), less likely to receive healthcare when needed (Kasman and Badley, 2004), and that people with one or more chronic conditions report less access to healthcare (Kontopantelis et al., 2010; Parchman et al., 2005; Wilson and Rosenberg, 2004). They also report poor self-perceived health (Ramage-Morin and Gilmour, 2010; Tripp et al., 2006) and various other physical and psychological problems (Millar, 1996; Ohayon and Schatzberg, 2010; Ramage-Morin, 2008; Smith et al., 1999; Toblin et al., 2011; Tsang et al., 2008). Furthermore, patients with different chronic illnesses have higher expectations, needs, and priorities of care, which may result in lower satisfaction when they do not receive the expected service (Jayasinghe et al., 2008).

The findings of this study also revealed that respondents’ self-reported overall health status was positively related to their assessment of the KPIs of healthcare in Alberta. This is consistent with existing studies showing that better self-reported overall health is associated with reports of higher satisfaction scores as well as better access to healthcare services (Cohen, 1996; Crow et al., 2002; de Boer et al., 2010; Hall et al., 1990, 1993; Jaipaul and Rosenthal, 2003; Jayasinghe et al., 2008; Põlluste et al., 2012; Rahmqvist and Bara, 2010; Ren et al., 2001; Sofaer and Firminger, 2005; Wendt et al., 2009; Xiao and Barber, 2008). Furthermore, results of this study indicated that respondents’ specific reports of poor physical and mental health status (during the past 30 days prior to the survey) were negatively related to their assessment of the KPIs of healthcare. This finding is also in agreement with previous studies reporting that patients who are in poor physical and mental health are less satisfied with the care that they receive (Al-Mandhari et al., 2004; Bleich et al., 2009; Glynn et al., 2004; Hall et al., 1999; Lyratzopoulos et al., 2012; Schoenfelder et al., 2011; Tucker, 2002; Wensing et al., 1997; Zhang et al., 2007).

One of the interesting findings of this study was that respondents’ knowledge of available health services strongly and positively predicted their assessment of the KPIs of healthcare in Alberta. However, it remains unclear as to why knowledge of health services emerged as one of the strongest predictors of the KPIs because no such indication can be found in existing literature. We could speculate that if people have good knowledge of the healthcare services at their disposal, they can make informed decisions about their health and become familiar with the availability of and accessibility to appropriate healthcare services in their community, which in turn would enable them to feel that they receive better care when they need it.

Overall, this study indicated that old age and chronic health problems (particularly chronic pain) predicted people’s assessment of the KPIs of healthcare in Alberta, net of socio-demographic factors, self-reported health statuses, and knowledge and utilization of health services. The findings suggested that people’s personal health and demographic characteristics are important factors associated with their perception of the healthcare system in the province. One of the key strengths of this study is that the KPIs of healthcare have been assessed in a representative sample of Albertans rather than only among patients enrolled in a medical care facility; the latter is the case in most of the studies conducted in this line of research. Another strength of this study is the use of multiple indicators (i.e., availability, accessibility, quality, and satisfaction) to measure the performance of healthcare in Alberta. Finally, the results implied that people experiencing chronic health problems may need healthcare services that are more responsive to their needs.

### Limitations

The findings of this study should be interpreted with caution due to several limitations. First, the findings are based on a self-report survey which may be subjected to over-reporting or underreporting by the respondents. For instance, Voaklander et al. (2006) suggested that discrepancies may exist between self-reported health survey responses and patients’ medical chart based information, such that some types of disease are more likely than others to be over-reported or underreported. However, given that the findings of this study are mostly consistent with existing literature, ‘courtesy bias’^a^ (Glick, 2009) is unlikely to be the case. Furthermore, most self-report surveys used in healthcare settings have been shown to be reliable. For instance, Raina et al. (1999) analyzed the reliability of several scales used frequently in population-based health surveys in Canada (in a group of seniors), and found that most of the multiple-item scales had acceptable internal consistency (Cronbach’s alpha ≥ 0.70), and most of the single-item measures also had acceptable test-retest reliability (kappa > 0.80).

Second, measures of the KPIs are based on several single-item questions capturing aspects of five indicators of healthcare, and as such, they may be subjected to over-simplifying complex issues and not addressing the full range of participants’ concerns about the performance of the health system in Alberta. However, Zhang et al. (2007) showed that a survey instrument based on single-item questions may be useful for examining patient satisfaction, self-rated health, and health confidence in primary care settings.

Third, the data did not allow for measuring the specific problems or experiences of dissatisfaction with healthcare services among the respondents. It has been noted that global surveys of patient experience generally present an overly optimistic view of the quality of care provided and do not identify areas of poor care (Staniszewska and Henderson, 2005). Therefore, future research, employing in-depth interviews or focus groups, should look into patients’ experiences of problems encountered while dealing with the healthcare system in order to identify areas for improvement.

Fourth, this study is cross-sectional in nature, and as such, does not permit inferring causal relationships among the variables, and the results have limited generalizability. To that end, more research is needed modeling longitudinal aspects of people’s assessment of their healthcare system. Fifth, it was not possible to control other socio-economic characteristics such as marital status, employment status, and racial and ethnic composition for present analysis because data were not available. Hence, future studies should control these factors in relation to people’s assessment of healthcare in Alberta. Finally, this study did not measure the time lag between respondents’ encounter with the healthcare system and answering the survey questions, a factor that may lead to differing assessment (Jackson et al., 2001).

### Implications

This study has several policy implications. A specific implication of this study is that the healthcare system in Alberta should provide services that are better tailored to the needs of people who experience chronic health problems. The facilitation of such support would most likely lead to increased patient satisfaction and better evaluation of the KPIs of healthcare in the province. As public assessment of performance indicators of healthcare is gaining momentum (Zhang et al., 2007), there is a need for taking into account people’s experiences in identifying potential areas of improvement in order to increase the effectiveness of healthcare systems (Hekkert et al., 2009). This can help physicians and healthcare providers to identify problems related to access, availability, quality, and outcome of care, and satisfaction with the care received from public’s point of view. Studies found that healthcare employees considered patients’ evaluations of their care useful for quality improvement (Heje et al., 2011; Iversen et al., 2010). In addition, a majority of healthcare professionals reported that they had implemented improvement measures by attending to problems identified by patients (Heje et al., 2011; Iversen et al., 2010).

## Endnotes

^a^A situation whereby respondents are reluctant to express negative opinions to an interviewer, leading to overestimation of satisfaction with healthcare services (Glick, 2009).

## Electronic supplementary material

Additional file 1: **Variable coding for the 2004 Alberta Health Survey.** (DOCX 12 KB)

## Abbreviations

KPIs: Key performance indicators.
