# Supplementary material for: Public assessment of key performance indicators of healthcare in a Canadian province: the effect of age and chronic health problems
Source: Springerplus. 2014 Jan 15;3:28. doi: 10.1186/2193-1801-3-28 (PMC3895437; doi:10.1186/2193-1801-3-28)
Supplement: Supplementary file 1 — Additional file 1: Variable coding for the 2004 Alberta Health Survey. (DOCX 12 KB) [file 40064_2013_775_MOESM1_ESM.docx]

| **Additional file 1** |  |
| --- | --- |
| **Variable coding for the 2004 Alberta Health Survey** | |
| Characteristics | Coding |
| Sex | 0 = Male, 1 = Female |
| Age | 0 = Non-seniors (18-64), 1 = Seniors (65-75+) |
| Education | Dummy coded for each of the 4 categories |
| Household Income | Dummy coded for each of the 4 categories |
| Living Status | 0 = Living alone, 1 = Living with someone |
| Self-reported Health status | 1= Poor, …. 5 = Excellent |
| Chronic Health problem | 0 = No, 1 = Yes |
| Received Health care services | 0 = No, 1 = Yes |
| Knowledge of the available Health services | 1= Poor, …. 4 = Excellent |
| Availability of Health care services | 1= Poor, …. 4 = Excellent |
| Accessibility of Health care services | 1= Very difficult, …. 4 = Very easy |
| Perceived Quality of Health care services | 1= Poor, …. 4 = Excellent |
| Quality of Health care system in Alberta | 1= Poor, …. 4 = Excellent |
| Satisfaction with the Health system in Alberta | 1= Very dissatisfied, …. 5 = Very satisfied |
| Days in past 30 days Physical health not good | Number of days |
| Days in past 30 days Mental health not good | Number of days |
| Own level of need for health services past year | 1 = Low, …. 3 = High |
